# Charting Recent Progress: Advances in Alcohol Research

**Published:** 1997

**Keywords:** research, AODE (alcohol and other drug effects), AODR (alcohol and other drug related) disorder, epidemiology, hereditary factors, environmental factors, brain, AOD use behavior, animal model, adverse drug effect, prenatal alcohol exposure, congenital anomaly, safety, economic cost of AODU (alcohol and other drug use), prevention of AODR problems, treatment, health services research, literature review, government agency

## Abstract

*The* Ninth Special Report to the U.S. Congress on Alcohol and Health *summarizes recent findings from a wide range of alcohol-related research areas. This article provides an overview of the topics covered in the* Ninth Special Report *and highlights some of the recent findings revealed through the application of innovative research methods*.

The *Special Report to the U.S. Congress on Alcohol and Health* is updated and submitted to Congress by the Secretary of Health and Human Services (HHS) every 3 years. This important document summarizes recent advances in alcohol research and charts current progress toward preventing and decreasing alcohol abuse and alcoholism. As well as being submitted to Congress, the report is distributed throughout the country to researchers, educators, treatment specialists, and other professionals interested in the current state of alcohol research.

Alcohol research has greatly increased our understanding of the biological, environmental, and social factors that contribute to alcohol use, abuse, and dependence as well as alcohol’s effects on both individuals and society. This diverse body of research—spanning disciplines that include biology, sociology, and economics—has advanced through the application of innovative research methods and techniques.

The *Ninth Special Report to the U.S. Congress on Alcohol and Health*, published in June 1997, focuses on research findings reported since the 1994 publication of the *Eighth Special Report*. In his introduction to the report, National Institute on Alcohol Abuse and Alcoholism (NIAAA) Director Enoch Gordis explains how specific areas of alcohol research have advanced through the application of new theories and techniques. For example, the application of neuroscience to the study of drinking behavior and addiction has enabled researchers to learn more about alcohol’s effects on the brain and to study the mechanisms of these effects in new ways.

This article briefly reviews the topics covered in the *Ninth Special Report* and highlights areas in which new research techniques are being applied. The remaining articles in this issue of *Alcohol Health & Research World* discuss how innovative research methods are being applied to specific areas of alcohol research, which may or may not be described in the *Ninth Special Report*.

## Epidemiology

Alcohol epidemiologists collect and analyze data about the rates of alcohol use, abuse, and dependence as well as other alcohol-related problems, including morbidity and mortality. Using a variety of data sources, including alcohol sales reports, U.S. vital statistics, hospital records, and surveys, researchers track alcohol consumption rates and the problems that can occur with drinking.

Average annual alcohol consumption per person began to decline in the early 1980’s and continued to drop through 1993, when it reached 2.25 gallons of alcohol—the lowest level recorded since 1964. National survey data also reveal increases in overall abstention rates and decreases in rates of heavy (i.e., five drinks on one occasion at least once per week) and weekly drinking. Factors thought to contribute to the decline in per capita consumption include less tolerant national attitudes toward drinking, increased societal and legal pressures and actions against drinking and driving, and increased health concerns among Americans.

Despite the declines in alcohol consumption, however, alcohol-related morbidity and mortality remain significant problems in the United States. Alcohol is a factor in an estimated 44 percent of all traffic crash fatalities. Furthermore, young people continue to be overrepresented in alcohol-related traffic fatalities. In 1993 drivers ages 16 to 24 accounted for 28 percent of all drinking driver deaths but represented only 15 percent of all licensed drivers in the United States.

Liver cirrhosis, another significant cause of alcohol-related deaths, was responsible for more than 25,000 deaths in the United States in 1992. Although the risk of demise from alcohol-related cirrhosis remains greater for men than for women and greater for blacks than for whites, cirrhosis death rates have decreased steadily among both men and women as well as whites and blacks since 1973.

In addition to morbidity and mortality, abusive drinking can also lead to legal, social, and job-related problems. Survey data suggest that despite decreases in alcohol consumption, specific drinking patterns, and cirrhosis mortality, the overall rates of dependence symptoms and social consequences did not change between 1984 and 1990. Among young people ages 18 to 29 who had never married and who were unemployed, however, significant increases in reports of two or more social consequences of alcohol abuse were found between 1984 and 1990.

Formal diagnostic systems have been developed to diagnose the alcohol use disorders known as alcohol abuse and alcohol dependence. The *Diagnostic and Statistical Manual of Mental Disorders* (DSM) is one such system. The most recent edition (i.e., the *Diagnostic and Statistical Manual of Mental Disorders, Fourth Edition* [DSM–IV]) defines alcohol dependence as a cluster of symptoms that includes continued drinking despite significant alcohol-related problems. Alcohol abuse is defined as repeated drinking in harmful situations with negative consequences. Data from a large 1992 U.S. household survey indicated that approximately 7.4 percent of the people sampled could be classified, according to DSM–IV criteria, as engaging in alcohol abuse, experiencing alcohol dependence, or both. Prevalence rates were higher for men than for women as well as for younger respondents compared with older ones.

## Influences on Alcohol Use and Abuse

The factors that influence drinking behavior are diverse and complex. Researchers have identified, however, the basic environmental and genetic components that contribute to a person’s overall risk for alcohol use and abuse. Research has shown, for example, that alcoholism runs in families and that genetic factors contribute substantially to a familial vulnerability for the disease.

Alcoholism is influenced by many genes located in different areas of a person’s DNA. Molecular geneticists are now using a group of techniques known as positional cloning in an attempt to identify the specific genes that contribute to a person’s risk for alcoholism. This effort is currently aimed at identifying the relevant chromosomes where the genes may be located. Researchers have also identified genes affecting alcohol elimination (i.e., metabolism) and have shown how these genes influence drinking behavior among certain Asian populations.

Through the use of animal models, researchers are investigating genetically influenced biological traits and behaviors—including alcohol consumption and preference, alcohol sensitivity, and alcohol metabolism—that are believed to be similar to aspects of human alcoholism. These traits are called quantitative traits, and a section of DNA thought to influence a quantitative trait is known as a quantitative trait locus (QTL). Once a QTL has been identified, a gene can be isolated and studied. This process has been used in mice to identify the possible location of genes that influence alcohol consumption, alcohol-induced hypothermia (i.e., reduced body temperature in response to alcohol), and other alcohol-related responses.

In addition to genetic influences, both individual and environmental factors contribute to drinking behavior and alcoholism risk. Researchers are currently exploring the influence of individual-level factors, such as alcohol sensitivity, physiological processes in the brain, personality characteristics, and cognitive processes.

A person’s sensitivity to alcohol has been found to be a heritable risk factor for alcoholism and a good predictor of who does and who does not develop alcoholism. People who are less sensitive to alcohol’s effects may be at relatively high risk for alcoholism because they lack effective feedback mechanisms that signal overconsumption.

The search for biochemical markers that can predict alcoholism has led researchers to examine the role of the serotonin neurotransmitter system (a key factor in brain cell communications) and the enzyme monoamine oxidase (MAO). MAO is involved in the breakdown of the neurotransmitters dopamine, serotonin, and norepinephrine, which have been linked to the risk for alcoholism. Although some studies have suggested that low MAO activity is an inherited risk factor for alcoholism, other studies have found no such association.

Research suggests that two broad dimensions of personality are associated with the risk for alcoholism. One such personality dimension, known as behavioral undercontrol, behavioral disinhibition, or deviance proneness, is marked by unconventionality, over-activity, aggression, and impulsivity. The second personality dimension associated with alcoholism risk has been termed “negative emotionality” and is marked by anxiety and depression. Researchers studying the cognitive factors that influence alcohol use have focused on the effects of alcohol expectancies (i.e., people’s beliefs about the anticipated consequences of drinking). Research has shown that alcohol expectancies differentiate alcoholics from nonalcoholics, are strongly correlated with current and future drinking, and appear to develop before exposure to alcohol.

Environmental factors that influence alcohol use include cultural norms, social influences of family and friends, and stressful life events. Cultural norms affect personal attitudes about the propriety of drinking and whether, how much, and when a person drinks. Drinking is also influenced by friends’ and family members’ drinking habits, parental standards regarding drinking, and the extent to which a person has been exposed to or is experiencing psychological stress.

## Alcohol’s Actions on the Brain

New research techniques have proved instrumental in advancing the study of the mechanisms by which alcohol produces intoxication, dependence, withdrawal, and other changes mediated through the central nervous system. Alcohol interacts with and alters the activities of many different cellular components, including cell membranes, proteins on the surface of nerve cells (i.e., neurons) that recognize neurotransmitters (i.e., receptors), chemicals that carry messages within cells (i.e., intracellular signaling enzymes), and genes. Because cell membranes are permeable to alcohol, the proteins within neurons are vulnerable to alcohol’s effects. Among the proteins affected by alcohol are the receptors for several neurotransmitters, including gamma-aminobutyric acid (GABA), glutamate, serotonin, and adenosine triphosphate (ATP).

Alcohol’s actions on GABA and glutamate receptors contribute to effects of intoxication including sedation, incoordination, and anxiety reduction. Some of alcohol’s effects through GABA receptors may involve the alteration of the receptor by the enzyme called protein kinase C (PKC). When PKC alters GABA receptors, their sensitivity to alcohol increases. Researchers have studied this result using the gene knockout technique, an experimental approach that involves inactivating genes in mouse cells. Using gene knockout mice, researchers have showed that deleting PKC abolishes alcohol-induced enhancement of GABA receptor activity. One type of glutamate receptor, the *N*-methyl-d-aspartate (NMDA) receptor, may play a role in alcohol-related blackouts and other alcohol-associated memory disorders.

The ATP receptor and a type of serotonin receptor known as 5-HT_3_ are also sensitive to alcohol. The 5-HT_3_ receptor has been implicated in alcohol’s intoxicating and addictive effects. In addition, alcohol may produce intoxication and dependence in part through direct or indirect effects on opioid receptors.

Within neurons, alcohol may disrupt multiple elements of intracellular communication. For example, acute alcohol exposure enhances the stimulation of adenylate cyclase, an enzyme necessary for normal cell function. Chronic alcohol exposure, however, produces a broadly suppressive effect on the stimulation of adenylate cyclase by multiple neurotransmitters.

Alcohol also influences PKC functions. For example, chronic alcohol exposure may increase the abundance and activity of PKC and result in a variety of adaptive changes. In addition, alcohol stimulation of PKC may contribute to alcohol-associated brain injury. PKC is responsible for many cellular activities, including protein synthesis and the actions of various membrane proteins. Therefore, alcohol-induced alterations in PKC activity can contribute to a wide range of adaptive responses within the central nervous system.

Alcohol’s effects on certain genes and gene products (i.e., protein synthesis) may promote some of the more enduring alcohol-associated changes in nerve cell function. Among the alcohol-responsive genes that have been identified are those for molecular chaperones, proteins that regulate the trafficking of other proteins within cells. Alcohol also increases the production of the gene responsible for tyrosine hydroxylase, an enzyme critical for the synthesis of the neurotransmitters dopamine and norepinephrine. Further research on these and other alcohol-responsive genes will provide clues about persistent alcohol-related changes in the central nervous system.

## Neurobehavioral Effects of Alcohol Consumption

Recent neuroscientific research has greatly expanded current knowledge of the brain structures and functions affected by alcohol. This knowledge has subsequently advanced our understanding of the links between alcohol’s effects on the brain and alcohol’s effects on behavior. Scientists have developed several animal models and advanced behavioral and biochemical tests to study alcohol-seeking behavior and the genetic and environmental influences that motivate and reinforce drinking. Animals selectively bred for alcohol preference or nonpreference have been used in various research approaches, including operant models, conflict tests, and drug discrimination procedures.

Researchers have used operant models—in which animals can obtain alcohol only by performing a specific task—to study the “cost” of alcohol (i.e., the amount of “work” required to obtain the drug) as an environmental influence on alcohol intake. These studies have shown that the amount of alcohol consumed can be modified by its cost, regardless of genetic preference for alcohol. These studies have also revealed an association between genetic predisposition for high alcohol intake and a greater motivation to work for alcohol.

Conflict tests, in which animals trained to respond to alcohol as a reinforcing stimulus are occasionally punished (e.g., by the administration of an electric shock), have been used to study alcohol’s anxiety-reducing properties. Such tests have shown that the stress-reducing actions of alcohol likely reinforce its continued use and contribute to the development of alcohol dependence.

Drug discrimination procedures determine the ability of animals trained to recognize alcohol to differentiate between alcohol’s subjective effects (e.g., lowered anxiety and increased euphoria) and the effects of other psychotropic drugs. In such tests, the identification of drugs that can partially or fully substitute for alcohol, combined with knowledge of the neurotransmitter systems affected by those drugs, helps researchers identify the brain pathways that mediate alcohol’s behavioral effects. Studies using drug discrimination procedures have shown that alcohol’s subjective effects are not mediated by any one neurotransmitter system but depend on the combined actions of multiple systems.

Among the neurotransmitters identified as mediators of alcohol-related behaviors are dopamine, serotonin, GABA, opioids, glutamate, and a new class of compounds known as neurosteroids. Dopamine has been implicated in both the reinforcement of alcohol-seeking behavior and the aversive effects of alcohol withdrawal.

Serotonin has long been associated with alcohol-seeking behavior in experimental animals and humans. Selectively bred alcohol-preferring rats have reduced serotonin content and fewer serotonin neurons in areas of the brain relevant to drug reinforcement than do non-alcohol–preferring rats. In humans, alcoholism is associated with a loss of serotonin neurons and deficiencies in serotonin synthesis, metabolism, and receptor function.

Receptors for GABA and glutamate appear to play important roles in the reinforcing actions of alcohol. Neurosteroids bind to and modulate the activity of both GABA receptors and the glutamate receptor NMDA. Some neurosteroids that act on the GABA receptor produce anxiety-reducing and hypnotic effects, whereas other neurosteroids can induce anxiety and convulsions. In addition, neurosteroids can enhance or interfere with the effects of glutamate by acting on the NMDA receptor.

Research involving selectively bred animals, advanced behavioral analyses, and new biomedical tests have helped advance the study of how the diverse behavioral effects of alcohol may be linked to alcohol-induced changes in the brain. Continued research aimed at identifying brain regions, receptor types, and interactive effects of neurotransmitter systems affected by alcohol is needed to better understand the mechanisms underlying alcohol abuse and alcoholism.

## Effects of Alcohol on Health and Body Systems

Excessive alcohol consumption can cause widespread damage to all tissues and organs of the body. Because the liver is the primary site of alcohol metabolism, damage to the liver may be among the most serious consequences of alcohol abuse. Alcohol has also been implicated in heart disease, neuropsychological disorders, endocrine system disruption, and immune system suppression.

Heavy alcohol use may cause liver inflammation and progressive liver scarring, leading to cirrhosis. Among the mechanisms thought to cause alcohol-related liver damage are the production of free radicals (i.e., products of alcohol metabolism that can interact with vital cell components such as proteins and DNA) and the release of cytokines (i.e., substances with inflammatory, scarring [i.e., fibrogenic], and growth-promoting properties). Acetaldehyde, a principal product of alcohol metabolism, can attach to cellular proteins, causing direct damage to liver cells or stimulating inflammatory autoimmune reactions in liver tissues. These changes may also stimulate liver cell collagen synthesis, a process thought to contribute to fibrosis and cirrhosis. A promising approach to preventing fibrosis involves the administration of polyunsaturated soybean lecithin, which may promote the breakdown of hepatic collagen.

Heavy drinking (i.e., consuming more than four drinks per day) has been associated with a variety of harmful effects on the heart and circulatory system. Long-term heavy alcohol use can disrupt the mechanical functions of the heart and may cause progressive functional changes and tissue damage, leading to heart disease and heart failure. Excessive drinking is also associated with high blood pressure and an increased risk for coronary heart disease and stroke. Light to moderate alcohol use, however, appears to help prevent coronary artery disease, a condition characterized by insufficient blood supply to the heart.

Prolonged alcohol use is associated with brain damage and the development of neuropsychological disorders. Among the impairments associated with alcoholism are deficits in short-term memory; disrupted cognitive and motor functioning; poor attention span; and difficulties with abstraction, problem-solving, and learning new information. Brain imaging techniques have demonstrated that alcoholism causes structural brain changes. For example, magnetic resonance imaging (MRI) and computed tomography (CT) allow the brain to be viewed inside the skull. Functional brain imaging techniques, such as positron emission tomography (PET), detect variables such as the regional distribution of blood flow and glucose metabolism within selected areas of the brain. Electrophysiological techniques, such as event-related potentials (ERP), measure the electrical activity of groups of nerve cells in various parts of the brain. In studies of alcoholics, these procedures have provided evidence of abnormal brain functioning and brain shrinkage. Researchers are using imaging techniques, along with neurocognitive tests, to identify connections between alcohol-associated structural changes in the brain and alcohol-associated cognitive impairment.

Alcohol use has also been found to interfere with normal endocrine system development and function. Research suggests that chronic alcohol use can disrupt the secretion of growth hormone, which may cause a variety of other metabolic and endocrine changes. In addition, alcohol abuse has been associated with sexual dysfunction in both men and women. Hormonal changes attributable to alcohol abuse have been linked to diminished libido, impotence, testicular degeneration, and decreased fertility in men. In nonalcoholic men, acute, low-dose alcohol intake has been found to lower serum testosterone levels. In women, the frequency of menstrual disturbances, spontaneous abortions, and miscarriages increases with the level of drinking, and alcohol abuse has adverse effects on fertility and sexual function. Alcohol use may also increase estrogen levels in women, an effect that may be related to an observed association between drinking and an increased risk for breast cancer. Alcohol’s adverse effects on reproductive development have also been demonstrated in animals: Chronic alcohol exposure in prepubertal rats, for example, has been shown to delay the onset of puberty.

Alcohol use, especially abusive or chronic use, depresses the immune system by disrupting the function, regulation, and distribution of immune cells. Prenatal and early postnatal exposure to alcohol can also disrupt immune system development. Suppressed immunity can cause increased susceptibility to bacterial infections, infectious disease, and cancer. Long-term alcohol abuse in humans can also contribute to autoimmune processes that damage liver tissues.

## Effects of Alcohol on Fetal and Postnatal Development

Prenatal alcohol exposure can cause a variety of harmful effects in the exposed fetus, ranging from a characteristic pattern of observable birth defects and mental impairment to more subtle cognitive and behavioral dysfunctions. Fetal alcohol syndrome (FAS) is the most severe birth defect resulting from prenatal alcohol exposure. People who exhibit some of the attributes of FAS but do not fulfill the diagnostic criteria for this condition are described as having “alcohol-related birth defects” (ARBD) or “fetal alcohol effects” (FAE). Although standardized criteria have been developed to diagnose FAS, clinicians and researchers have difficulty identifying people with this condition. Perhaps the greatest problem in diagnosis is that none of the characteristic abnormalities are specific to FAS. An otherwise healthy person may display one or two of the diagnostic traits. The characteristic facial abnormalities can be subtle and difficult to recognize, and they can change as a person ages.

Researchers are evaluating the use of computer-assisted techniques to aid in FAS diagnosis. Using a computer-based analysis of facial photographs of 7-year-old children who were exposed prenatally to heavy drinking, researchers have been able to quantitatively define the characteristic FAS face of this age group. Other approaches that may improve FAS diagnosis include the development of a specific behavioral profile for people affected by prenatal alcohol exposure and the use of brain imaging techniques to detect markers of alcohol-induced injury.

Studies among adolescents and adults with FAS suggest that the deficits associated with the disorder are pervasive and enduring. Although many of the physical characteristics associated with FAS become less prominent as the affected person reaches puberty, intellectual problems persist, and behavioral, emotional, and social problems become more pronounced. Children with FAS and ARBD are often described as being hyperactive and impulsive and having short attention spans. Behavioral problems, such as poor judgment, failure to consider the consequences of one’s actions, and difficulty perceiving social cues are common, even among children who are not considered retarded according to IQ scores.

The relationships between the quantity, frequency, timing, and pattern of maternal drinking and the outcomes observed in children exposed to alcohol prenatally have been addressed by studies that track such children over time. Some of these studies have found an association between prenatal alcohol exposure and growth deficits at birth. Slower growth has been found to persist in 6- to 8-month-old infants and in children up to age 6. These studies have also revealed attention deficits, slower reaction times, and poorer academic performance among alcohol-exposed children.

Research has not yet fully explained how the amount and timing of alcohol exposure and maternal drinking patterns disrupt particular stages of fetal development. Several studies have found that maternal drinking during the first trimester is associated with facial anomalies in children. Alcohol-induced neurobehavioral effects may also be related to certain periods of exposure. Heavy maternal drinking during the first and second trimesters, for example, seems to increase the occurrence of delayed language development in children. Studies assessing the relationship between the amount of maternal drinking and fetal effects suggest that physical development is disrupted at a considerably higher dose of alcohol than is cognitive function.

Because alcohol-related birth defects are entirely preventable with maternal abstinence from alcohol during pregnancy, prevention is an important research issue. Researchers are attempting to develop multilevel strategies that consider the many factors that influence drinking in different racial, ethnic, and socioeconomic groups. These strategies include community education to heighten awareness of the risks of drinking during pregnancy, approaches to effectively identify women whose drinking places them at risk for adverse pregnancy outcomes, and strategies aimed at intervening with individual women who are problem drinkers and thus at greater risk for prenatally exposing their children to alcohol.

Animal models of alcohol-induced birth defects have been important tools in the study of the risk factors, mechanisms, and consequences related to prenatal alcohol exposure. Animal models allow researchers to conduct controlled experiments of how alcohol acts on a fetus, how other influences associated with alcoholism in humans contribute to the adverse outcomes of prenatal exposure to alcohol, and how FAS and ARBD can be treated or prevented. Such experiments have shown that peak maternal blood alcohol concentration (BAC) determines the likelihood and severity of alcohol-induced birth defects. Therefore, both the total amount of alcohol consumed and the pattern of drinking are important factors that influence fetal outcomes. In addition, animal studies have demonstrated that the period(s) during pregnancy when alcohol concentrations are high has an important influence on the development of birth defects associated with prenatal alcohol exposure.

Animal studies of the effects of prenatal alcohol exposure on the developing brain show that such exposure can cause dramatic anatomical changes in the fetal central nervous system by altering cell growth, migration, and differentiation. The amount, duration, pattern, and timing of exposure can influence specific neuroanatomical outcomes. These studies also show that prenatal alcohol exposure affects the development and function of neurotransmitter systems in the brain, including the serotonin, dopamine, acetylcholine, glutamate, and GABA systems.

## Effects of Alcohol on Behavior and Safety

A large body of research has shown that alcohol use and abuse both directly and indirectly influence a variety of human behaviors with potentially serious consequences. More than 100,000 deaths annually in the United States result from alcohol-related causes, including traffic crashes, falls, fires, and drownings. Drinking also has been associated with high-risk sexual behavior, family and marital violence, homicide, physical assault, and other criminal behaviors.

Alcohol and injury may interact in two ways. First, the context and place in which drinking occurs may contribute to an increased risk for injury. For example, drinking in bars, where the risk of assault may be high, likely exposes a person to more hazardous circumstances than does drinking at home. Alcohol may also contribute to injury through direct biological effects on perception and responsiveness to potential hazards. Data on alcohol-related nonfatal injuries come largely from emergency room studies, which provide information about the types of injuries treated, patients’ BAC’s, patients’ demographic characteristics, and self-reported drinking before and after an injury. Coroner reports are the main source of information on alcohol-associated fatalities and can be used to compare alcohol involvement in different types of injuries and across demographic characteristics.

Although a large proportion of traffic crash fatalities involve alcohol, this rate is declining. From 1983 to 1993, the proportion of fatal crashes that involved alcohol decreased 26 percent. The number of drivers ages 16 to 24 killed in alcohol-related traffic crashes decreased by 40 percent between 1977 and 1993. This decline is attributed to the adoption of legislation in 1977 that raised the minimum legal drinking age to 21. Between 1977 and 1993, the number of male drivers of all ages involved in alcohol-related crashes decreased 22 percent, whereas the number of female drivers involved in such crashes increased by 18 percent. Despite this trend, the total number of fatal traffic crashes involving women drivers who were legally intoxicated (i.e., having a BAC of 0.10 or greater) has remained far below that of men.

Direct links between alcohol and other types of injuries, such as those associated with aircraft crashes, fires and burns, boating accidents, and violence, are less clear. Simulated flight experiments have documented alcohol’s adverse effects on pilots’ planning, performance, and vigilance, but the epidemiological evidence for alcohol’s role in airplane crashes is limited.

Alcohol use may be a significant risk factor in drownings and in other fatal or nonfatal injuries that occur on or near water. Drinking has been shown to affect a person’s ability to operate vessels and boating equipment and to contribute to physiological changes that reduce the probability of survival in or near water. Boat passengers who drink alcohol are also at increased risk of injury, regardless of the drinking behavior of the boat operator. Because alcohol impairs balance and motor function, a passenger could be at risk for falling overboard, even when the boat is drifting or being operated safely. Alcohol may also contribute to diving accidents that result in spinal cord injury.

Alcohol use may contribute to injuries caused by burns and fires by causing drowsiness and increasing the risk of falling asleep while smoking. The effects of alcohol may also reduce awareness of smoke and fire alarms and may impair one’s ability to escape from a burning building by increasing disorientation associated with smoke and panic. In addition, drinking may affect the outcome of burn injuries. Burn patients with positive BAC’s have been shown to have higher fatality rates than patients without detectable blood alcohol.

As stated earlier, a strong relationship has been found between alcohol and various types of violence, including homicides, suicides, and spousal abuse. Internal processes, such as biological impulses, may interact with alcohol to predispose a person to violence. External events, including situational factors, are thought to stimulate cognitive mechanisms that influence alcohol-related violence. These mechanisms may involve expectations about alcohol’s effects or changes in perception and cognitive processing that accompany alcohol consumption.

A review of studies examining the link between drinking and homicide found that in most cases, more than 60 percent of homicide offenders were drinking at the time of the offense. One study of the association between alcohol and suicide found that nearly 36 percent of suicide victims had a positive BAC.

Alcohol is also present in a substantial proportion of domestic violence incidents. Researchers have suggested that alcohol contributes to aggression by increasing sensitivity to pain and frustration. In addition, alcohol may impair problem-solving ability through effects on the brain’s frontal lobe. People given alcohol have been shown to display increased sensitivity to electric shocks compared with persons given a placebo. Furthermore, intoxicated persons exposed to a frustrating event have been found to experience stronger frustration and react with more aggression than sober people exposed to the same event.

Drinking has also been associated with risk-taking and sensation-seeking behavior among both adolescents and adults. Several researchers have suggested that drinking, risk taking, and sensation seeking may be part of a broader personality style of behavioral undercontrol. People with such traits may be more likely to participate in various alcohol-related risky behaviors, such as drinking and driving and unprotected sexual activity. Risky driving, including drinking and driving, is more prevalent among younger than older drivers. Research suggests that drinking and driving may be part of a larger constellation of problem behaviors in young adulthood. The prevalence of drinking and driving among high school students was found to increase significantly with the frequency of drinking and with the frequency of binge drinking. In addition, the prevalence of drinking and driving was found to be higher among students who used alcohol and other drugs compared with those who only used alcohol.

Drinking has been associated with high-risk sexual behavior among both young people and adults. Alcohol has disinhibiting effects that may increase the likelihood of unprotected sex, which in turn can facilitate the spread of the human immunodeficiency virus (HIV) and other sexually transmitted diseases. Research has also found that people who met the diagnostic criteria for alcohol abuse or alcohol dependence or who were heavy or binge drinkers had an increased risk of exposure to HIV and of developing AIDS.

## Economic Aspects of Alcohol Use and Alcohol-Related Problems

Recent research has explored the influence of economic factors on alcohol consumption and on various alcohol-related problems. For example, researchers studying the determinants of alcohol consumption have concluded that the consumption of beer, wine, and distilled spirits declines in response to increases in the prices or taxes associated with these beverages. Other studies in this area suggest that drinkers with the highest consumption levels may be much less sensitive to price changes than are drinkers who consume at more moderate levels. Research also indicates that increases in alcoholic beverage taxes and prices are associated with reductions in motor vehicle fatalities, especially among young drivers.

Studies of the influence of alcohol advertising on drinking and alcohol-related problems have produced inconsistent findings. Although some recent studies found no significant link between advertising and consumption levels, one investigation found that increases in alcohol advertising could be linked to increases in traffic fatality rates.

Research on the economic aspects of alcohol use and abuse has also examined the effects of drinking and problem drinking on the labor market. Studies of the effects of alcohol consumption on earnings have found that both heavy drinkers and people who abstain from alcohol have lower earnings than do people who drink moderately. Alcohol abuse and alcohol dependence, however, have generally been found to have pronounced negative effects on earnings and income levels. Recent research suggests that alcohol problems may have indirect effects on earnings and employment by influencing educational achievement and marital status. For example, frequent drinking in high school has been associated with fewer years of college completion.

Substantial progress has been made in recent years toward specifying and estimating important policy-relevant economic relationships. Recent methodological advances, such as theories of advertising behavior and improved statistical and analytical techniques, have allowed more ambitious research in these areas. The use of new data sources will allow more detailed study of these issues and more meaningful economic analysis of the behaviors associated with alcohol use and abuse.

## Prevention of Alcohol Problems

The prevalence of social and health-related problems associated with alcohol use highlights the need for prevention strategies. Prevention researchers target individual drinkers as well as social and physical environments in which drinking occurs. Individual-level approaches aim to change people’s beliefs or attitudes about alcohol, strengthen individual psychological and social abilities, or restructure environments to reduce the risk for alcohol-related problems.

Many individual-level programs are school-based, because schools offer access to a large target audience of current and potential young drinkers. Such programs aim to decrease the prevalence and level of drinking among youth, with the goal of preventing alcohol abuse later in life. Over the past 20 years, researchers have developed a variety of school-based programs, many of which were found to have limited effectiveness. More recent programs, however, designed and implemented with greater scientific rigor and improved methodologies, have demonstrated promising results. For example, the Alcohol Misuse Prevention Study (AMPS), a program designed to help young people resist factors leading to alcohol consumption, was shown to be especially effective for high-risk sixth-graders. The positive effects of AMPS, which persisted through grade 12, were produced partly by reducing adolescents’ vulnerability to peer pressure.

Young people have consistently been found to believe that alcohol use is more prevalent among their peers than it actually is. Programs designed to correct these misperceptions have been useful in reducing drinking among youth. Students exposed to this type of education within the Adolescent Prevention Trial, a program that compared this strategy with training to resist peer pressure, had significantly lower alcohol use than those exposed to other components in the program.

Environmental-level prevention efforts either consider the physical and social factors regulating exposure to alcohol or mediate the risk that drinking poses to a person. Such approaches include warning labels, strategies that seek to limit both alcohol availibility and drinking and driving, and community-based prevention programs.

Beginning in 1989 warning labels were required by law for all alcoholic beverage containers. Studies have shown that awareness of the warning labels increased over time and that regular drinkers were more likely than other drinkers to be aware of the labels. The labels were found to have only modest effects on risk perceptions and drinking practices, however. Further research has suggested that more prominent placement and stronger wording of the warnings may improve both users’ awareness and the overall effectiveness of the labels.

Interventions and policies that affect alcohol availability may alter levels of alcohol consumption and alcohol-related problems. Several studies have shown that when States enact policy changes that eliminate alcohol retail monopolies and introduce licensed private sales outlets, the sales and consumption of alcohol increase substantially. Privatizing alcohol distribution systems often alters alcohol availability by increasing the number of stores, lengthening sale hours, and increasing advertising and promotion.

Laws establishing 21 as the minimum legal drinking age were designed to limit alcohol availability to youth. Although many underage youth still drink, research has shown that these laws have been effective in reducing youth drinking and related problems, such as traffic crashes.

Various prevention strategies have been developed to target drinking and driving, a problem that contributes to about 45 percent of all traffic crashes in the United States. These strategies include legal approaches, such as lower allowable BAC’s for young drivers, and voluntary approaches, such as free taxis and designated-driver programs. Lowering the allowable BAC for young drivers was found to result in a 16-percent decline in the proportion of fatal single-vehicle nighttime crashes among young drivers. The Wichita Ride Service Program, which provided free taxi rides during the winter holiday season to visibly intoxicated persons, was found to effectively deter potentially impaired drivers. One evaluation of the designated driver strategy found that fraternity members who agreed to appoint designated drivers often forgot to do so and when they did, the designated drivers typically drank alcohol, although less than they usually would.

Communitywide prevention programs combine multilevel prevention approaches to address a range of populations and risk behaviors. Such programs target the social, political, and economic factors that contribute to problem drinking. Some communitywide programs have been found to effectively increase awareness, public support for alcohol prevention, and related attitudes. For many of these programs, however, changes in drinking behavior or in the prevalence of alcohol-related problems appear to be temporary.

## Treatment of Alcoholism and Related Problems

Considerable progress has been made in recent years in several areas of alcoholism treatment. In 1994 the American Psychiatric Association published its revised diagnostic manual, DSM–IV, which includes substantive changes in the diagnostic criteria for alcohol dependence and alcohol abuse. Current research on the impact of these changes and comparisons with other widely used diagnostic systems will provide practical information to guide clinical decisions as well as government and insurance company policies concerning reimbursement for different types of treatment.

Formal diagnosis of alcohol use disorders includes patient assessment, which involves the use of interviews to determine characteristics that may affect treatment choice and treatment prognosis. In biomedical research, a new emphasis has been placed on the patient’s quality of life as an outcome measure for treatment. This new focus has been applied in the alcoholism treatment field through the use of specialized assessment instruments that measure intra- and interpersonal functioning and related areas pertinent to alcoholism recovery. Among the factors thought to be important predictors of treatment success or failure are subjective well-being; drinking-related beliefs; patient readiness to change; and alcohol-related expectancies, social functioning, and social support for drinking or abstinence.

A treatment approach known as brief intervention is currently being offered to people who are alcohol abusers but do not meet the criteria for alcohol dependence. Brief intervention involves the early detection of harmful substance use before any physical dependence has developed. This approach differs from most alcohol treatments for several reasons. Brief intervention generally is restricted to four or fewer sessions and is usually performed in a treatment setting not specific for alcoholism—typically a primary health care setting. In addition, it is usually performed by personnel who are not specialists in addiction treatment. The goal of the intervention—which generally consists of short, face-to-face conversations between the patient and a health care worker or counselor—may be moderate drinking rather than abstinence. Research shows that brief interventions can be effective, but further evaluation is needed to identify the types of patients best suited for this treatment method.

Progress has continued in the development of pharmacological treatments for alcoholism. Medications being tested for alcoholism treatment include detoxification agents to manage alcohol withdrawal, alcohol-sensitizing agents to deter patients from drinking, and anticraving agents to reduce the hunger for alcohol and the risk of relapse to drinking.

Interventions to prevent relapse to drinking are an important component of treatment. Research suggests that positive and negative expectancies about the effects of alcohol are important influences in posttreatment drinking decisions and that expectancies should be addressed in treatment.

The appropriateness of controlled drinking—an approach that limits drinking rather than insists on abstinence—as a therapeutic goal for alcoholism treatment remains highly controversial in the United States. A number of patient characteristics influence whether this strategy is appropriate, including severity of dependence, extent of drinking history, psychological dependence, previous treatment, and current liver damage. Research has demonstrated successful maintenance of controlled drinking by a small subset of treated patients. Those patients with less severe dependence symptoms and those who reject the label of alcoholic and the goal of abstinence at treatment outset seem most likely to be successful in achieving long-term asymptomatic drinking.

## Alcohol Health Services Research

Alcohol health services research examines the effectiveness of alcoholism treatment and prevention in real-world settings. This research effort evaluates the influences of individual, organizational, and financing factors on access and cost-effectiveness of treatment. The goal of this emerging area of research is to improve the accessibility, quality, effectiveness, and cost-effectiveness of prevention and treatment. Alcohol health services research is taking place within a context of dramatic shifts in the organization and financing of health care in general. This state of flux presents difficult methodological and theoretical challenges. Most published work in this area is based on research conducted before changes occurred in the health care system and provides baseline information that will help researchers assess new approaches.

Many studies have focused on the availability of alcohol treatment services, both generally and to different population groups. Based on data from the National Drug and Alcoholism Treatment Utilization Survey, the number of alcohol-only and alcohol and other drug treatment facilities increased by nearly 150 percent between 1982 and 1993. From 1982 to 1992, the percentage of programs offering specialized services for women also increased.

Among the factors shown to influence entry into treatment are gender, age, marital status, and ethnicity. For women, factors predicting entry into treatment include being older, unmarried, and white and having a lower level of education, employment, and income. For men, the relevant factors include having experienced alcohol-related social consequences, being older, and belonging to an ethnic minority. Access to treatment may not be equal across ethnic groups. For example, national data show that the percentage of Hispanics in public alcohol treatment programs is lower than the overall rate of drinking problems in that population, whereas the percentage of African-Americans in such programs is higher than the rate of alcohol problems in that group.

Managed care has limited treatment costs by restricting unnecessary care. Initially, managed care affected only private treatment. Recently, State and county systems, as well as public insurance programs, have begun to adopt similar approaches. In addition, managed care organizations have made a pronounced effort to reduce the use of inpatient services because they are more expensive than outpatient services.

Cost-effectiveness is an important issue in alcohol health services research, but only a few studies have addressed this topic. One study has suggested that brief motivational counseling was the most cost-effective treatment modality among several approaches that were evaluated and that aversion therapy was among the least cost-effective. Several investigations have shown that outpatient treatment is at least as effective as inpatient treatment. Further research on matching treatment to need, on treatment effectiveness, and on evaluating different organizational systems for structuring treatment will help guide the development of future Advances in Alcohol Research services and establish priorities for insurance coverage.

## Conclusion

The *Ninth Special Report to the U.S. Congress on Alcohol and Health* summarizes the current state of alcohol research and presents the progress that has been made in understanding alcohol abuse and alcoholism since the 1994 publication of the *Eighth Special Report*. Research across a broad range of disciplines continues to improve our understanding of the factors that contribute to alcohol use, abuse, and dependence as well as the effects that alcohol use has on both individual people and society. Further research advances in these areas will lay the foundation for future investigation into the causes, treatment, and prevention of alcohol-related problems.

## Ordering Information

The *Ninth Special Report to the U.S. Congress on Alcohol and Health* is sold for a cost-recovery fee of $11, including shipping and handling, within the continental United States. The order form for the *Ninth Special Report* can be downloaded from NIAAA’s Internet Web site (http://www.niaaa.nih.gov).

